# Prosthetic rehabilitation of an edentulous patient with microstomia using digital and conventional methods: A case report

**DOI:** 10.1002/ccr3.7904

**Published:** 2023-10-25

**Authors:** Naeime Moslemian, Mahya Hasanzade

**Affiliations:** ^1^ Department of Prosthodontics, School of Dentistry Hamedan University of Medical Sciences Hamedan Iran; ^2^ Department of Prosthodontics, Dental Research Center Dentistry Research Institute, Tehran University of Medical Sciences Tehran Iran

**Keywords:** artificial teeth, complete denture, dental impression technique, microstomia, mouth abnormalities

## Abstract

This study describes prosthetic rehabilitation an edentulous patient with microstomia. Maxillary preliminary and definitive impression were made by intraoral scanning and custom 2‐piece impression tray respectively to fabricate conventional denture.

## Graphical abstract

Maxillary sectional trays for final impression with a locking mechanism using magnet.

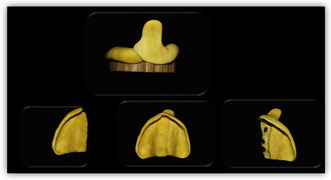

## 1 INTRODUCTION

Microstomia, defined as an abnormally small oral orifice, can occur as a result of trauma including facial tissue injuries, animal bites, electrical and thermal lesions, and chemical burns.^1^, ^2^ This condition can also arise from genetic disorders, diseases such as Plummer–Vinson syndrome, and the collagen group of diseases including submucosal fibrosis and scleroderma^2^ or surgical treatment for orofacial cancers and reconstruction of lip defects.^3^ The main problems due to microstomia can be of functional (speech, nutrition, and hygiene) or esthetic (due to asymmetric lip placement) groups. Many treatment options, including the surgical, nonsurgical, and a combination of these two, are available to cope with this abnormality. The aim of treatment is not only providing well‐functioning lips with increased mouth opening and esthetic improvement but also providing a stable and long‐lasting result.^4^ Some techniques such as surgery and denture design modification have been recommended for problem management associated with providing dental prostheses for edentulous patients with microstomia.^5^ Making high‐quality preliminary impressions which record all anatomic landmarks is mandatory for the successful complete denture treatment. During the impression procedures in patients with microstomia, wide opening of the mouth is required for the proper tray insertion and alignment, but this is impossible.^6^ Preliminary impressions from patients with microstomia have been made using various tray modifications such as modified stock trays, flexible trays, or without conventional ones using an initial polyvinyl siloxane (PVS) impression. Although these techniques have been successfully implemented, the proper method for making a preliminary impression has not been determined. Nowadays, using computer‐aided technologies make possible to design and fabricate complete denture. Making impressions with intraoral scanners could eliminate tray selection and adaptation, infection transmission from patients, and impression transference to the laboratory.^7^ However, several factors such as saliva reflection and dynamic movements of soft tissues make the digital impression from edentulous arch difficult. Making final impression in these patients needs alternative techniques like the sectional impression trays that has been recommended that trays should be cut into two unequal sections so that the labial frenum is recorded accurately in the impressions.^8^ The two halves of the tray could be joined using LEGO building blocks, fins in the handle, metal pins, and burs.^6^ Similarly, removable complete and partial dentures have employed the sectional or collapsible designs to retain them in an unfolded position. The corresponding examples include the insertion of pins, use of a locking tool, latching a swing‐lock assembly, locking the denture segments with magnets or attachments and also using flexible denture materials.^8^ It is impossible to make a collapsible maxillary denture consisting of only two sections because of the palatal vault. Therefore, a third section is usually used for this purpose.^3^

The aim of this clinical report is to present the prosthetic treatment applied to an edentulous patient with microstomia using an intraoral scanner for preliminary impression and fabricating complete removable prostheses.

## 2 CASE HISTORY

**FIGURE 1:**
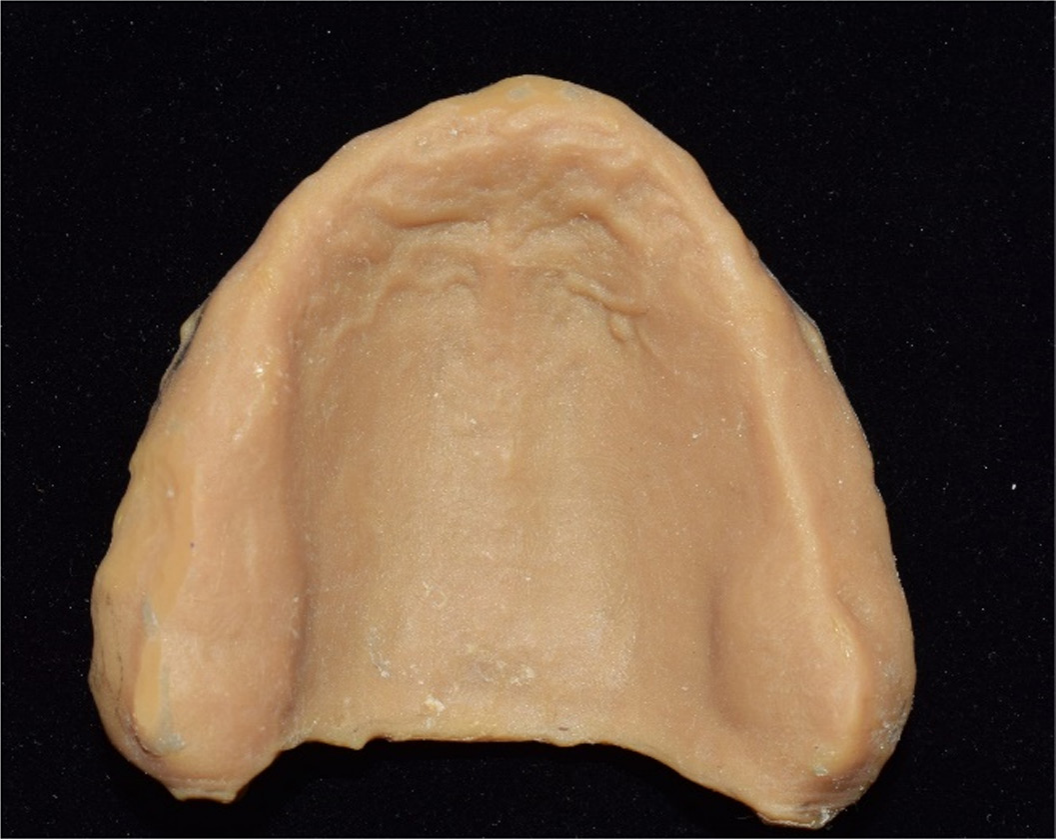
Printed maxillary cast.

A 45‐year‐old woman with microstomia was referred to the prosthodontic department for receiving complete denture for both maxillary and mandibular arches in June 2018. She had scar tissues, burned face, and deformed hands caused by burning in an accident. The maximum oral opening was approximately 20 mm in height and 35 mm in width, with tight and inflexible labial tissues. The mandibular alveolar ridge was resorbed moderately. After discussion and offering various treatment options, the patient agreed to undergo surgical enlargement of the oral aperture and then fabrication of conventional complete denture. The preliminary impression making was started after 4 weeks of commissuroplasty. Petroleum jelly was used on the commissures. The preliminary mandibular impression was made with the smallest edentulous stock tray and irreversible hydrocolloid (Chromogel alginate; Marlic Medical Industeris Co.). The tray inserted by 90° rotation while an intraoral mirror was used for retracting the lips as much as possible. For the preliminary maxillary impression, stock trays with various sizes and shapes were examined. However, inserting them was not possible due to the limited mouth opening. Therefore, digital maxillary impression was taken using an intraoral scanner (TRIOS 3 Basic; 3shape). The retraction of lip and cheek and maxillary vestibular area stretching were performed by an intraoral scanner tip in order to successfully scan the soft tissues. The mandibular impression was poured in type II dental stone (Dental Plaster; Pars Dandan). The scan data were then converted to the standard tessellation language (STL) file and transported to 3D printing device (Digi Dent Plus; Mobtakeran Mecathronic ARK Co.) to print the model with resin (Freeprint model 2.0; Detax) with 25–100 μm accuracy (Figure 1). A sectional custom maxillary tray was fabricated using the autopolymerizing acrylic resin (Acrylic acropars; Marlic Medical Industeris Co.) on the printed model. Two sections of the tray were unequal with the right section being larger, crossing the midline, and extending to the left buccal frenum. Moreover, it had specified butt joint border on the outer surface and magnet attachment. However, the smaller tray section had specified butt joint border on the intaglio surface and magnet attachment on the outer side to be attached to the first one. In addition, a conventional custom mandibular tray was fabricated with the same acrylic resin. Tray borders were trimmed in order to have 2 mm space above the vestibular depth for the border molding.

**FIGURE 2:**
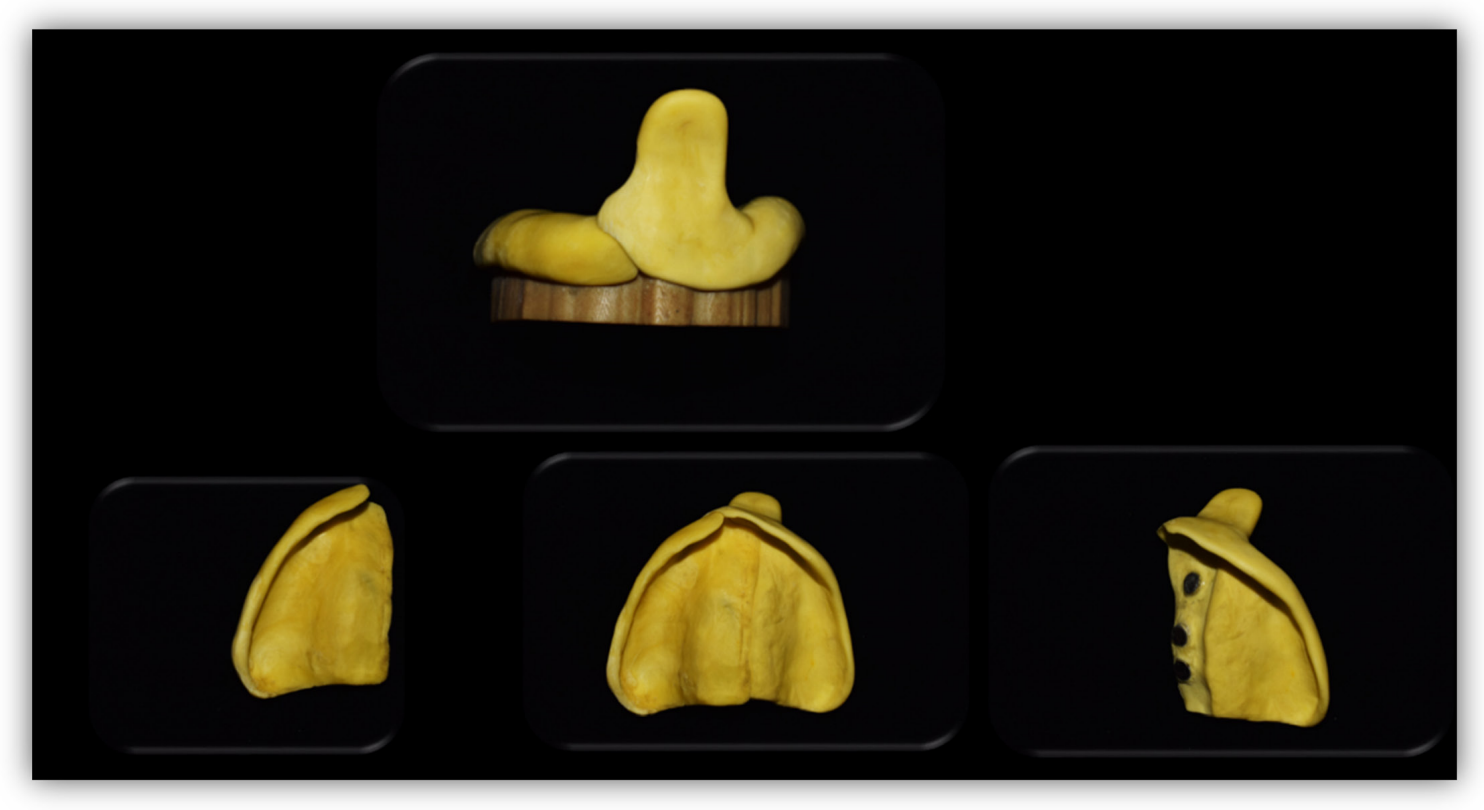
Maxillary sectional trays with a locking mechanism using magnet. Note the magnets that are on the inner surface of the bigger section and the outer surface of the smaller section.

**FIGURE 3:**
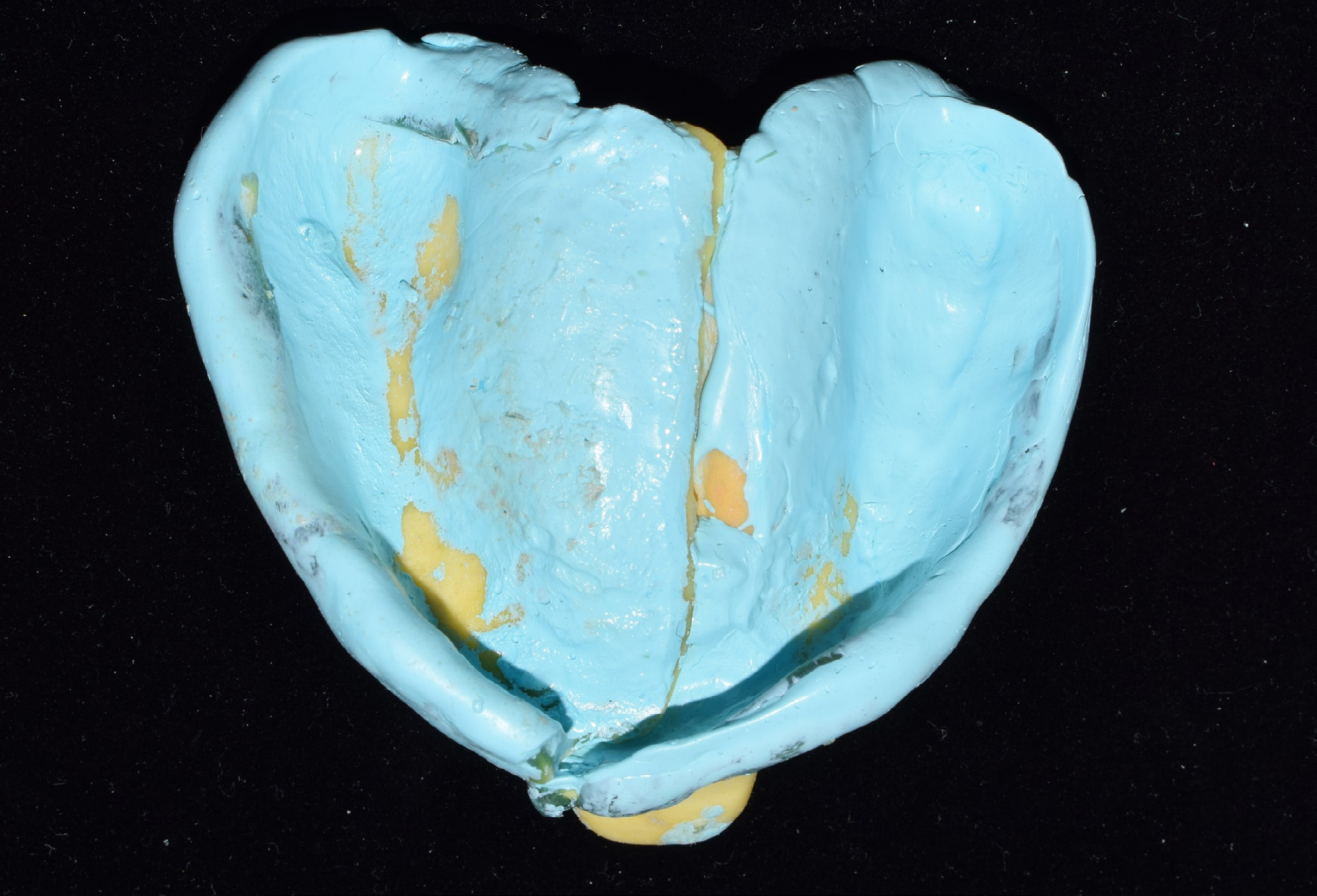
Maxillary final impression.

**FIGURE 4:**
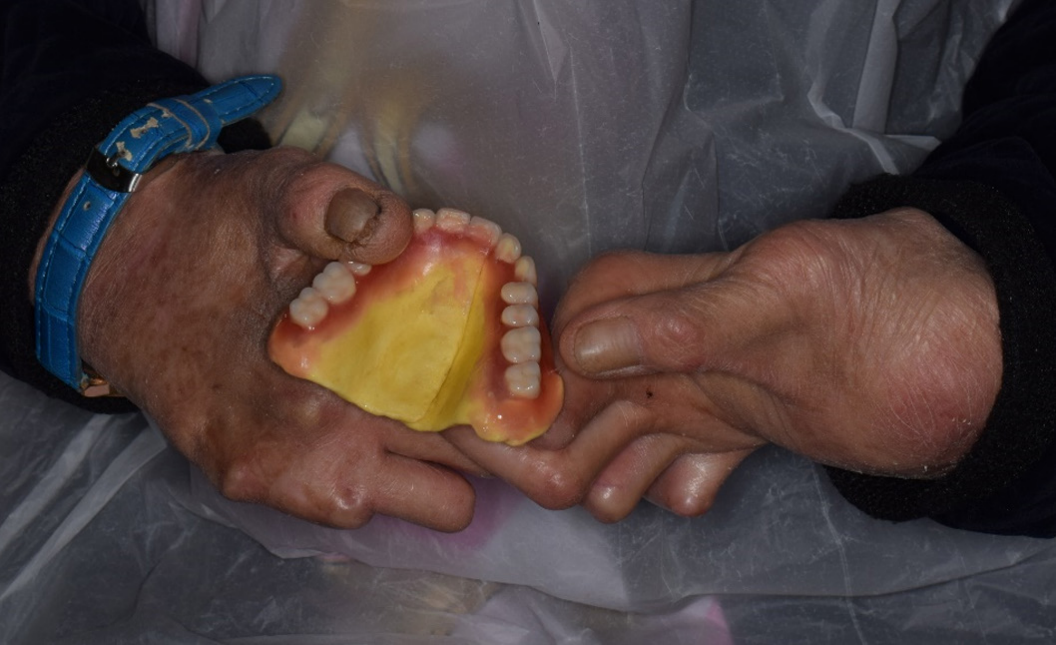
Hands deformity.

Labial and buccal vestibule, frena, and postpalatal seal areas were functionally recorded with modeling plastic impression compound (PERI Compound; GC, Japan) (Figure 2). The final impression was made with zinc oxide, eugenol‐free impression paste (ZOE) (Cavex outline; Cavex). The impression paste was initially placed in the larger tray segment and inserted in the mouth. Then, the other tray segment with the impression paste was placed over the magnets to ensure locking of the two tray segments. After the impression material set, the tray segments were removed from the mouth one by one and fixed together outside (Figure 3). The mandibular tray border was molded with thermoplastic sticks (Isofunctional; GC), and definitive impressions were made with ZOE (Cavex outline; Cavex). Final impressions were boxed and poured using ADA type III dental stone (Dental Plaster; Pars Dandan). The maxillary record base and occlusal rims were prepared in two pieces as the right and left segments being attached to each other by magnet. They were then placed in mouth and adjusted according to esthetic and phonetic. The maxillomandibular relationship was recorded in centric relation, and definitive casts were mounted on a semi‐adjustable articulator (Hanau H2; Whip Mix Corp.). The semi‐anatomic artificial teeth (A1, B13 Finex; Beta Dent) were arranged with bilateral balanced occlusion. Esthetic, phonetic, and occlusion were evaluated in try‐in session. The important point is that the patient could not assemble the segments because of hands deformity, and therefore, integrated maxillary denture was planned for the final prostheses (Figure 4). At the delivery appointment, denture base extensions were evaluated, excessive pressure of the intaglio surface was relieved, and the occlusion was adjusted to derive simultaneous tooth contact in centric and eccentric positions. The prosthesis placement was demonstrated to the patient and delivered as well. The hygienic recommendations were explained to the patient as well. The patient had no difficulties using the dentures, and satisfactory results were obtained during a 5‐year follow‐up period. The compatibility of the intaglio surface of the denture with the underlying tissue and occlusion was checked annually. Little discoloration was seen in the artificial teeth.

## 3 DISCUSSION

For the examined patient, the preliminary maxillary impression was made with intraoral scanner, which is a challenging issue in edentulous arches due to record displaceable soft tissues.^7^ During scanning procedure, there are some difficulties in capturing the frenula and vestibular sulcus in stitching separate pictures because of the insufficient landmarks such as remaining teeth and the great data file size after the completion of the edentulous maxillary arch scan.^9^ This method has some advantages in comparison to PVS without trays for the preliminary impressions. The weight of gypsum may be destroyed impression while pouring the cast, and imprecise cast may not reflect the soft tissues accurately that will have a negative effect on retention and stability of definitive prosthesis.^7^ Kim et al. applied intraoral scanning for definitive impression to a patient with an excessively tight reconstructed lip. Digitalization of the complete denture fabrication process can simplify the complicated treatment and laboratory process of the conventional methods.^10^ In our report, the intraoral scanning was only used for the preliminary impression, while sectional trays applied for definitive ones. This allowed lengthening the tray borders to the vestibular depth and functionally register peripheral tissues by border molding. Zinc oxide, eugenol‐free impression paste is used as the final impression material in order to facilitate assembling of tray sections after removing from the mouth. Saygili et al. used both digital and conventional techniques for the edentulous patient with microstomia. A collapsible denture base for the posterior region and a rounded triangular one for the anterior region with stud attachment were fabricated for the maxilla.^7^

In the present report, commissuroplasty surgical intervention and mouth stretching exercises, which have been recommended by Naylor,^11^ facilitated the treatment process. The patient's malformed hands did not allow the use of collapsible denture base. In the try‐in appointment, the patient was educated, and she was able to insert interim integrated denture bases in her mouth by rotating it 90°. Thus, conventional complete denture was fabricated as the definitive restoration. Integrated denture base found to have several advantages for the patient such as good retention and stability compared to the sectional dentures, easy manipulation particularly for patient with disabled hands, less price, and good maintenance. Accurate recording of mobile soft tissues for final impressions of edentulous arches by scanners will allow us to be a step closer to a fully digital, complete denture fabrication workflow.^7^

## 4 CONCLUSION

Adjunct therapies can be implemented prior to the fabrication of complete dentures in patients with microstomia. Intraoral scanners can be also used in patients with an excessive gag reflex, allergic reactions to the impression materials or microstomia.

## AUTHOR CONTRIBUTIONS

**Naeime Moslemian:** Methodology; project administration; writing – original draft. **Mahya Hasanzade:** Supervision; writing – review and editing.

## FUNDING INFORMATION

The authors received no financial support for the research, authorship, and/or publication of this article.

## CONFLICT OF INTEREST STATEMENT

The authors declare no conflict of interest.

## CONSENT

Written informed consent was obtained from the patient to publish this report in accordance with the journal's patient consent policy.

## Data Availability

Data available on request from the authors.
